# Maxillomandibular Advancement and Upper Airway Stimulation for Treatment of Obstructive Sleep Apnea: A Systematic Review

**DOI:** 10.3390/jcm11226782

**Published:** 2022-11-16

**Authors:** Ning Zhou, Jean-Pierre T. F. Ho, René Spijker, Ghizlane Aarab, Nico de Vries, Madeline J. L. Ravesloot, Jan de Lange

**Affiliations:** 1 Department of Oral and Maxillofacial Surgery, Amsterdam UMC, University of Amsterdam, Meibergdreef 9, 1105 AZ Amsterdam, The Netherlands; 2 Academic Centre for Dentistry Amsterdam (ACTA), University of Amsterdam and Vrije Universiteit Amsterdam, 1081 LA Amsterdam, The Netherlands; 3 Department of Orofacial Pain and Dysfunction, Academic Centre for Dentistry Amsterdam (ACTA), University of Amsterdam and Vrije Universiteit Amsterdam, 1081 LA Amsterdam, The Netherlands; 4 Department of Oral and Maxillofacial Surgery, Northwest Clinics, 1815 JD Alkmaar, The Netherlands; 5 Medical Library, Amsterdam UMC, University of Amsterdam, 1105 AZ Amsterdam, The Netherlands; 6 Cochrane Netherlands, Julius Center for Health Sciences and Primary Care, University Medical Center Utrecht, 3584 CG Utrecht, The Netherlands; 7 Department of Otorhinolaryngology—Head and Neck Surgery, OLVG, 1061 AE Amsterdam, The Netherlands; 8 Department of Otorhinolaryngology—Head and Neck Surgery, Antwerp University Hospital (UZA), 2650 Edegem, Antwerp, Belgium

**Keywords:** obstructive sleep apnea, therapy, maxillomandibular surgery, hypoglossal nerve, systematic review

## Abstract

This systematic review aimed to comparatively evaluate the efficacy and safety of maxillomandibular advancement (MMA) and upper airway stimulation (UAS) in obstructive sleep apnea (OSA) treatment. A MEDLINE and Embase database search of articles on MMA and/or UAS for OSA was conducted. Twenty-one MMA studies and nine UAS studies were included. All the MMA studies demonstrated a reduction in apnea hypopnea index (AHI) postoperatively, and success rates ranged from 41.1% to 100%. Ten MMA studies reported pre- and postoperative Epworth sleepiness scale (ESS), and all but one study demonstrated a reduction in ESS. In the UAS studies, all but one demonstrated a reduction in AHI, and success rates ranged from 26.7% to 77.8%. In the eight UAS studies reporting pre- and postoperative ESS, an ESS reduction was demonstrated. No studies reported any deaths related to MMA or UAS. The most common postoperative complications after MMA and UAS were facial paresthesia in the mandibular area and discomfort due to electrical stimulation, respectively. This systematic review suggests that both MMA and UAS are effective and generally safe therapies for OSA. However, due to the limitations of the included studies, there is no evidence yet to directly compare these two procedures in OSA treatment.

## 1. Introduction

Obstructive sleep apnea (OSA) is a prevalent sleep-related breathing disorder characterized by recurrent upper airway obstruction during sleep [1], and its overall prevalence ranges from 9% to 38% in the general adult population [2]. OSA is associated with considerable health risks, such as cardiovascular and cerebrovascular disease [3,4]. Continuous positive airway pressure (CPAP) is accepted as the first-line therapy for moderate to severe OSA, but poor compliance and suboptimal use of CPAP drive OSA patients to seek alternative therapies, including other non-invasive therapies and surgical treatment [5,6].

Moderate-to-severe OSA is usually caused by multilevel obstructions of the upper airway, which highlights the need for surgical therapies able to resolve multilevel upper airway collapse [7]. One such therapy that has existed for many decades is maxillomandibular advancement (MMA) [8,9]. MMA is a multilevel skeletal surgery in which the maxilla and mandible are advanced by a combination of a Le Fort I osteotomy of the maxilla and a bilateral sagittal split osteotomy of the mandible [8,9]. By expanding the skeletal framework attached with the pharyngeal soft tissues, MMA enlarges the velo-orohypopharyngeal airway [10] and increases the tension of the pharyngeal soft tissues, decreasing the collapsibility of the upper airway [11]. MMA is currently considered as the most effective surgical treatment modality for moderate-to-severe OSA in adults aside from tracheostomy.

A more contemporary therapy is hypoglossal nerve stimulation (HNS), which works by electrically stimulating the branches of the hypoglossal nerves that innervate muscles responsible for protruding the tongue and thus maintaining upper airway patency during sleep [12]. Currently, there are three different systems for HNS therapy, including the Aura6000 Targeted Hypoglossal Neurostimulation system (LivaNova PLC, London, England, UK), the GenioTM system (Nyxoah SA, Mont-Saint-Guibert, Belgium), and the Inspire II upper airway stimulation (UAS) system (Inspire Medical Systems, Maple Grove, MN, USA) [13]. Given that the Inspire UAS system is the most widely used system having Food and Drug Administration (FDA) approval for clinical use [14], this review only focused on UAS therapy (Inspire^®^ system). Over the past decade, UAS has emerged as an effective therapy and therefore has become an increasingly popular treatment option for moderate-to-severe OSA [15,16].

Currently, the main indications for MMA are moderate-to-severe OSA, and mild OSA in patients presenting with a dentofacial deformity [17]. UAS therapy is generally indicated for patients with the following characteristics: moderate-to-severe OSA (apnea hypopnea index (AHI) 15–65 events/h with <25% central or mixed apneas), positive airway pressure (PAP) therapy failure, and absence of complete concentric velum collapse (CCCp) on drug-induced sleep endoscopy (DISE) [18]. When no generally accepted indicative results are found during clinical, laboratory, or endoscopic examinations (e.g., significant skeletal-dental deformity, AHI > 65 events/h, CCCp on DISE), patients with moderate-to-severe OSA may be expected to benefit from MMA as well as UAS therapy. Although MMA and UAS have both demonstrated efficacy and safety for patients, there is a paucity of evidence on comparison of these two treatment options [17].

Therefore, the purpose of this systematic review is to comprehensively evaluate and compare the efficacy of MMA and UAS for moderate-to-severe OSA through the assessment of AHI and Epworth sleepiness score (ESS) as primary outcomes. Secondly, the postoperative complications of these two therapies were investigated.

## 2. Materials and Methods

This systematic review was performed in accordance with the preferred reporting items for systematic review and meta-analysis (PRISMA) statement [19]. The protocol for this systematic review was registered at PROSPERO (PROSPERO ID: CRD42021261394; https://www.crd.york.ac.uk/prospero/display_record.php?RecordID=261394 (accessed on 14 November 2022)).

### 2.1. Selection Criteria

The inclusion criteria were: (1) adult patients (> 18 years old) with moderate-to-severe OSA diagnosed by polysomnography (PSG; AHI ≥ 15 events/h); (2) patients who underwent MMA or UAS for OSA; (3) studies that reported pre- and postoperative PSG data; (4) studies with a follow-up ≥ 6 months; (5) study designs: randomized controlled trials (RCTs), quasi-experimental studies, and cohort studies; and (6) English language.

The exclusion criteria were: (1) sample size < 10 patients; (2) patients who underwent other adjunctive surgical procedures (e.g., uvulopalatopharyngoplasty) at the time of MMA or UAS; and (3) preliminary studies in which the findings had been nested in other studies with larger sample size and/or longer follow-up.

### 2.2. Literature Search

A literature search was performed with the help of an information specialist (RS) using MEDLINE and Embase databases on 14 December 2021. Search terms and search strategies used for each database are available in Supplementary Materials (Table S1 (a)).

### 2.3. Study Selection

After removal of duplicate articles, the remaining results were screened based on title and abstract by two independent reviewers (NZ and JH). The full texts of potentially relevant articles were retrieved and further evaluated by NZ and JH independently for compliance of studies with the eligibility criteria. Discrepancies were resolved by discussion. Reference lists of eligible studies were checked for additional studies.

### 2.4. Data Extraction

The extracted data included article title, year of publication, first author, study design, specific surgical technique, length of follow-up, sample size, age, gender, body mass index (BMI), preoperative and postoperative PSG data (AHI, respiratory disturbance index (RDI), and oxygen desaturation index (ODI)), preoperative and postoperative ESS score, preoperative and postoperative data on quality of life (QoL), surgical success rate and cure rate, and postoperative complications. According to the accordion severity grading system of surgical complications [20], the postoperative complications were classified as major or minor depending on the needs for endoscopic or interventional radiologic procedures or reoperation as well as failure of one or more organ systems.

Data were extracted by NZ and JH independently. Discrepancies were resolved through discussion. If RDI was reported by a study, it would be extracted as AHI, since these two respiratory parameters have been consolidated based on the 2013 American Academy of Sleep Medicine’s manual for the scoring of sleep and associated events [21]. If there were multiple follow-up data in a study, the data with longest follow-up time were included. Surgical success was defined as “a postoperative AHI < 20 and at least 50% reduction in AHI after surgery” [22], and surgical cure was defined as “a postoperative AHI < 5” [23].

### 2.5. Quality Assessment

Methodologic quality assessment of each study was performed by NZ and JH independently, and any discrepancies were resolved by discussion.

The Methodological Index for Non-Randomized Studies (MINORS) quality assessment tool, a validated tool for the methodological assessment of non-randomized surgical studies [24], was used to assess the methodological quality of the included studies. The MINORS tool is composed of eight items applicable to all non-randomized studies and four additional items specifically for comparative studies. Each item was scored as 0 (not reported), 1 (reported but inadequate), or 2 (reported and adequate), giving a global ideal score of 24 for comparative studies and 16 for non-comparative studies. For comparative studies, the categorizations are as follows: 0–6, very low quality; 7–10, low quality; 11–15 fair quality; and ≥16, high quality. For non-comparative studies, the categorizations are as follows: 0–4, very low quality; 5–7, low quality; 8–12, fair quality; and ≥13, high quality [25].

### 2.6. Statistical Analysis

The collected parameters (age, BMI, AHI, ODI, and ESS) were pooled by weighted average and weighted standard deviation [26]. When there were RCTs or comparative studies between MMA and UAS, meta-analyses were performed to compare the overall effect of MMA and UAS in treating OSA. Heterogeneity of the studies was assessed using the I^2^ statistic with a cutoff of 25% (low), 50% (moderate) and 75% (high) [27]. When moderate-to-high heterogeneity was present, a random effects model was adopted; otherwise, a fixed effects model was used. Because some patients may report multiple complications, the complication rate of each study was calculated by dividing the number of events by the number of patients.

## 3. Results

### 3.1. Search Results

**Figure 1:**
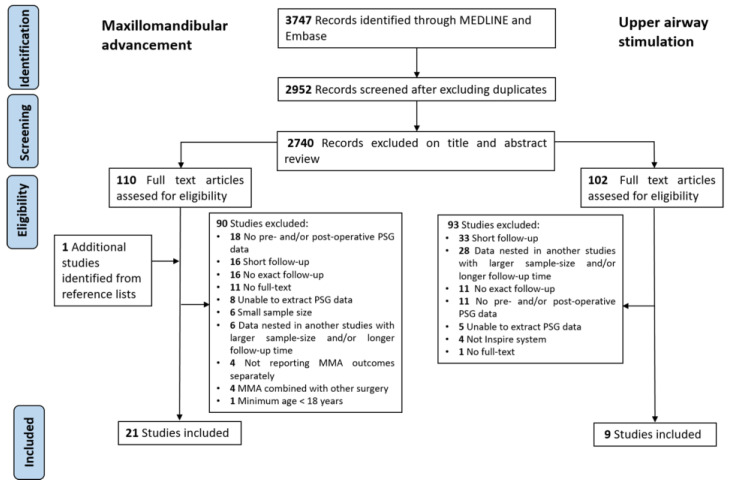
PRISMA flow diagram of the study selection process.

The flow diagram of study selection progress is summarized in Figure 1. A total of 2952 studies were screened after deduplication, and 212 were retrieved for full-text review. 

MMA group. Twenty-one studies [11,28,29,30,31,32,33,34,35,36,37,38,39,40,41,42,43,44,45,46,47] were identified, producing a pooled data set of 581 patients (male 78.5%) with a weighted age of 42.2 ± 11.5 years and a weighted BMI of 28.1 ± 6.4 kg/m^2^. The mean follow-up period from surgery to final postoperative PSG was 25.9 months (range, 6 months–12.5 years). One study [39] was excluded from the analyses for clinical efficacy because the data of a subset of the patients with a longer follow-up period were nested in another included study [38]. The characteristics of these studies are shown in Table 1.

UAS group. In total, nine studies [15,48,49,50,51,52,53,54,55] were identified, yielding 1029 patients (male 96.2%) with a weighted age of 55.1 ± 10.1 years and a weighted BMI of 29.1 ± 4.2 kg/m^2^. The mean follow-up period was 18.8 months (range, 6 months–5 years). The characteristics are summarized in Table 2.

Because there was no RCT or comparative study of MMA and UAS in treating OSA, a meta-analysis could not be performed to compare their overall effect sizes on OSA.

**Table 1:** Characteristics of studies on maxillomandibular advancement.

Study	Design	N	Age (Years) (Mean ± SD)	% Male	Degree of Advancement (mm) (Mean ± SD)		Follow-Up (Mean ± SD)	BMI (Mean ± SD)		AHI (Mean ± SD)		ODI (Mean ± SD)		ESS (Mean ± SD)		% Success	% Cure
					Max	Mand		Pre-op	Post-op	Pre-op	Post-op	Pre-op	Post-op	Pre-op	Post-op		
Bettega et al., 2000 [28]	Retro	20	44.4 ± 10.6	90	11.8 ± 0.5	11.8 ± 0.5	6 m	26.9 ± 4.3	25.4 ± 3.3	59.3 ± 29.0	11.1 ± 8.9					75 ^c^	
Bianchi et al., 2014 [29]	Retro	10	45 ± 14	100	10	10	6 m			56.8 ± 5.2	12.3 ± 5.5						
Boyd et al., 2015 [30]	Pro	14			7.0 ± 2.3	9.2 ± 3.3	6.6 ± 2.8 y			50.0 ± 20.0	8.0 ± 10.7						
Conradt et al., 1997 [31]	Retro	15	44 ± 12	93.3			>2 y	28.3 ± 3.4		51.4 ± 16.9	8.5 ± 9.4						
Gerbino et al., 2014 [32]	Pro	10	44.9		9.2 ± 1.2	10.4 ± 2.2	6 m	31.6 ± 5.5	28 ± 1.4	69.8 ± 35.2	17.3 ± 16.7	59.5 ± 5.3	9.1 ± 8			80 ^d^	
Goh et al., 2003 [33]	Pro	11	42.8 ± 8.2	100	10	10	7.7 m	29.4 ± 4.6	27.2 ± 3.3	70.7 ± 15.9	11.4 ± 7.4					81.8	
Goodday et al., 2016 [34]	Retro	13	37.8 ± 8.6	84.6			9.6 m	38.8 ± 10.9	37.3 ± 8.0	117.9 ± 9.2	16.1 ± 26.2			12.9 ± 5.5 ^b^	5.0 ± 4.1 ^b^	76.9	46.2
Hsieh et al., 2014 [35]	Pro	16	33 ± 7.9	75			12 ± 8 m	22 ± 3.3		35.7 ± 18	4.8 ± 4.4					100	
Kastoer et al., 2019 [36]	Pro	14	51.1 ± 7.3	57.1			6 m	25.7 ± 3.7		40.2 ± 25.6	9.9 ± 7.2	13.5 ± 18.6	4.0 ± 3.5	13 ± 6	9 ± 7		
Li et al., 1999 [39]	Retro	175	43.5 ± 11.5	83			6 m			72.3 ± 26.7 ^a^	7.2 ± 7.5 ^a^					95 ^e^	
Li et al., 2000 [38]	Retro	40	45.6 ± 20.7	82.5	10.8 ± 2.7	10.8 ± 2.7	4.2 ± 2.7 y	31.4 ± 6.7	32.2 ± 6.3	71.2 ± 27.0 ^a^	7.6 ± 5.1 ^a^					90 ^e^	
Li et al., 2001 [40]	Retro	52	46.6 ± 6.7	82.7	10.5 ± 1.5		6 m	32.0 ± 6.0		61.6 ± 23.9 ^a^	9.2 ± 8 ^a^					90 ^f^	
Li et al., 2002 [37]	Pro	12	47.3 ± 9.8	75	10.5 ± 1.2	10.5 ± 1.2	6 m	33.5 ± 6.2	32.3 ± 4.1	75.3 ± 26.4 ^a^	10.4 ± 10.8 ^a^					83.3 ^f^	
Liao et al., 2015 [41]	Pro	20	33.4 ± 6.5	85			14 ± 9.3 m	22.4 ± 3.4		41.6 ± 19.2	5.3 ± 4			11.9 ± 7.3	7 ± 3	100 ^c^	
Lin et al., 2020 [42]	Pro	53	35.7 ± 11.7	75.7	4.3 ± 2.9	13.3 ± 3.8	24 m	24.8 ± 3.3	23.9 ± 4.7	34.8 ± 26.0	7.4 ± 6.7			10.8 ± 5	10.2 ± 5.1		67.9
Liu et al., 2016 [11]	Retro	20	44 ± 12	85	7 ± 1.4		6 m	27 ± 4.6	27.4 ± 4.6	53.6 ± 26.6	9.5 ± 7.4	38.7 ± 30.3	8.1 ± 9.2	17.0 ± 4.8	5.7 ± 2.7	90	50
Rubio-Bueno et al., 2017 [43]	Pro	34	40.8 ± 13.9	41.2	4.9 ± 3.2	10.4 ± 3.9	6 m	27.6 ± 4.5	25.5 ± 4.3	38.3 ± 10.7	6.5 ± 4.3	34.7 ± 12.5	5.4 ± 4.1	17.4 ± 5.4	0.8 ± 1.4	100	52.9
Veys et al., 2017 [44]	Pro	10	44.7 ± 9.5	80	4.8 ± 2.8	8.3 ± 2.3	6 m			26.8 ± 12.7	12.3 ± 14.4			14.1 ± 5.9	5.7 ± 3.0	70	40
Vicini et al., 2010 [45]	RCT	25	49.1 ± 9.1	92		11	13 ± 2.5 m	32.7 ± 5.8	31.4 ± 6.5	56.8 ± 16.5	8.1 ± 7			11.6 ± 2.8	7.7 ± 1.3	88	36
Vigneron et al., 2017 [46]	Retro	29	40.7 ± 12.6		8.4 ± 4.1	11.7 ± 5.1	12.5 ± 3.5 y	24.6 ± 4		56.6 ± 24	25.5 ± 20.6				7.5 ± 4.7	41.4	
Wu et al., 2019 [47]	Retro	28	37.2 ± 11.8	53.6	2.0 ± 3.1	8.8 ± 3.7	>1 y	24.2 ± 5.1		59.3 ± 14.5	10.9 ± 3.3			12.8 ± 2.8	6.9 ± 2.5	85.7	46.4

AHI, apnea–hypopnea index (events/h); BMI, body mass index (kg/m^2^); ESS, Epworth sleepiness scale; m, months; Max, maxilla; Mand, mandible; N, number of patients; ODI, oxygen desaturation index (events/h); post-op, postoperative; pre-op, preoperative; pro, prospective; RCT, randomized controlled trial; retro, retrospective; y, years. ^a^ Respiratory disturbance index (RDI) in this study was extracted as AHI. ^b^ The number of patients was 9. ^c^ This study defined surgical success as an AHI < 15/h with ≥ 50% reduction in postoperative AHI. ^d^ This study did not define the criteria of surgical success. ^e^ This study defined surgical success as an RDI < 15/h with ≥ 50% reduction in postoperative RDI. ^f^ This study defined surgical success as a postoperative RDI < 20/h.

**Table 2:** Characteristics of studies on upper airway stimulation.

Study	Design	N	Age (Years) (Mean ± SD)	% Male	Follow-Up (Month)	BMI (Mean ± SD)		AHI (Mean ± SD)		ODI (Mean ± SD)		ESS (Mean ± SD)		% Success	% Cure
						Pre-op	Post-op	Pre-op	Post-op	Pre-op	Post-op	Pre-op	Post-op		
Bachour et al., 2021 [55]	Retro	15	52.9 ± 6.6	86.7	18 ± 9.6	29.1 ± 3.3	30.1 ± 4.5	33.0 ± 16.5	36.5 ± 23.8	25.3 ± 18.3	30.3 ± 21.1	11.5 ± 3.8	8.1 ± 4.5	26.7	6.7
Heiser et al., 2017 [48]	Pro	20	57 ± 12	100	12	28.1 ± 13.1		28.9 ± 7.6	6.6 ± 5.1						
Philip et al., 2018 [49]	Pro	10	52.0 ± 9.4	100	6	28.8 ± 3.3		46.7 ± 12.2	14.5 ± 8.9	38.1 ± 21.1	10.5 ± 9.9	15.9 ± 3.5	10.0 ± 6.1		
Steffen et al., 2019 [50]	Retro	18	51.5		24	27.9 ± 4.5	28.0 ± 4.7	26.3 ± 10.6	10.4 ± 10.1	12.8 ± 10.2	10.1 ± 12.0	12.7 ± 5.2	5.1 ± 3.8	77.8	33.3
Steffen et al., 2020 [51]	Pro	38	58.0 ± 10.0	97.4	36	29.1 ± 3.9	28.6 ± 3.3	30.0 ± 13.7	13.1 ± 14.1	25.8 ± 16.7	11.6 ± 14.0	12.1 ± 5.8	6.0 ± 3.2	62	35
Suurna et al., 2021 [54]	Pro	782			14.3 ± 7.0	29.2 ± 4		35.8 ± 15.0	14.5 ± 14.9			11.4 ± 5.5	7.1 ± 4.6	69.7	
Van de Heyning et al., 2012 [52]	Pro	28	55.1 ± 9.2	96.4	6	29.5 ± 2.5		42.3 ± 16.4	32.6 ± 29.1	30.7 ± 21.6	26.7 ± 27.0	11.0 ± 5.0	7.6 ± 4.3	50	
Vanderveken et al., 2013 [53]	Retro	21	55 ± 11	95.2	6	28 ± 2		38.5 ± 11.8	20.3 ± 20.6			8.2 ± 5.0 ^a^	6.4 ± 4.3 ^a^	62	
Woodson et al., 2018 [15]	Pro	97	54.4 ± 10.3		60	28.6 ± 2.5		30.4 ± 9.4 ^b^	12.4 ± 16.3	27.2 ± 10.0 ^b^	9.9 ± 14.5	11.3 ± 5.2	6.9 ± 4.7 ^c^	74.6 ^b^	44

AHI, apnea–hypopnea index (events/h); BMI, body mass index (kg/m^2^); ESS, Epworth sleepiness scale; N, number of patients; ODI, oxygen desaturation index (events/h); post-op, post-operative; pre-op, pre-operative; pro, prospective; retro, retrospective. ^a^ The number of patients was 18. ^b^ The number of patients was 71. ^c^ The number of patients was 92.

### 3.2. Quality Assessment

MMA group. One of the included studies was an RCT of MMA and autotitrating positive airway pressure (APAP), one was a retrospective quasi-experimental study, ten were prospective cohort studies, and nine were retrospective cohort studies. As only the MMA cohort of the RCT was included in the analyses, after omitting the unrequired APAP cohort, this study was regarded as a single-arm trial. The quality of the RCT was therefore assessed using the MINORS tool as per the other included studies. Of these studies, three studies were classified as “high quality”, and the others were classified as “fair quality” (Supplementary Table S2 (a)).

UAS group. Six prospective studies and three retrospective studies were included. Of these, one study was classified as “high quality” and eight studies as “fair quality” (Supplementary Table S2 (b)).

### 3.3. Respiratory Parameters

MMA group. Fifteen MMA studies [11,28,29,30,31,33,34,35,36,37,41,42,44,45,47] reported a significant reduction in AHI postoperatively (*p* < 0.05). The others [32,38,40,43,46] reported an AHI reduction but did not report a *p* value. All the studies [11,28,29,30,31,32,33,34,35,36,37,38,40,41,42,43,44,45,46,47], totaling 446 patients, demonstrated a weighted baseline AHI of 54.6 ± 27.4/h and a weighted postoperative AHI of 10.1 ± 10.8/h.

Of four studies [11,32,36,43] (n = 78) reporting pre- and postoperative ODI, two demonstrated a significant reduction in ODI after MMA (*p* < 0.05), and the other two also reported an ODI reduction but without a *p* value. The weighted pre- and postoperative ODIs were 35.1 ± 22.8/h and 6.3 ± 6.4/h, respectively.

UAS group. Of the selected studies, the study by Bachour et al. [55] did not show a significant reduction in AHI postoperatively. Five studies [48,49,50,51,54] demonstrated a significant reduction in AHI postoperatively (*p* < 0.05), and three studies [15,52,53] showed an AHI reduction but did not report a *p* value. The weighted pre- and postoperative AHIs in 1003 patients were 35.2 ± 14.7/h and 15.0 ± 16.1/h, respectively. 

Of six studies [15,49,50,51,52,55] reporting pre- and postoperative ODI, the study by Bachour et al. [55] did not find a significant improvement in ODI postoperatively, while the others [15,49,50,51,52] reported a reduction in ODI after surgery, of which two studies did not report a *p* value. The weighted pre- and postoperative ODIs were 26.5 ± 16.0/h and 14.6 ± 18.5/h (n = 180), respectively.

### 3.4. Subjective Parameters

MMA group. Of nine studies [11,34,36,41,42,43,44,45,47] (n = 217) reporting pre- and postoperative ESS, the study from Lin et al. did not show an improvement in ESS after MMA, one study demonstrated a reduction in ESS but without a *p* value, and the others reported a significant reduction in ESS (*p* < 0.05). The weighted pre- and postoperative ESS values were 13.1 ± 5.5 and 6.7 ± 4.8, respectively.

Three studies [30,42,44] assessed pre- and postoperative QoL. Boyd et al. found that after MMA, there was a significant improvement in the Functional Outcomes of Sleep Questionnaire (FOSQ) (*p* < 0.05) [30]. Veys et al. assessed the subjective outcome of MMA using the OSA QoL questionnaire. They found that there was an improvement in all of the following six symptoms after MMA—daytime sleepiness, snoring, concentration, waking up at night, headache, and high blood pressure—while the influence of MMA on nocturia and sexual activity was variable [44]. Lin et al. found that there was no significant improvement in Short Form-36 quality of life (SF-36) after MMA [42].

UAS group. Of eight studies [15,49,50,51,52,53,54,55] reporting pre- and postoperative ESS, seven demonstrated a significant reduction in ESS postoperatively (*p* < 0.05), and one reported a ESS reduction but did not report a *p* value. The weighted pre- and postoperative ESS values were 11.4 ± 5.4 (n = 1006) and 7.0 ± 4.6 (n = 1001), respectively.

Two studies reported pre- and post-UAS FOSQ scores. The STAR trial cohort demonstrated an increase in FOSQ score five years after surgery (14.3 ± 3.3 to 18.0 ± 2.2). Van de Heyning et al. also found a significant improvement in FOSQ score postoperatively (89.1 ± 23.5 to 100.8 ± 16.9, *p* < 0.05).

### 3.5. Surgical Success and Cure

MMA group. Surgical success rate of MMA was available in 15 studies [11,28,32,33,34,35,37,38,40,41,43,44,45,46,47] and ranged from 41.1% to 100%. Surgical cure rate of MMA was reported in seven studies [11,34,42,43,44,45,47] and ranged from 36% to 67.9%.

UAS group. Surgical success rate of UAS was available in six studies [15,50,51,52,54,55], ranging from 26.5% to 77.8%. Surgical cure rate was reported in four studies [15,50,51,55] and ranged from 6.7% to 44%.

### 3.6. Long-Term Follow-Up Outcomes

MMA group. Five studies [30,31,38,42,46] reported long-term follow-up (≥2 years) data in 151 patients with weighted baseline AHI of 51.7 ± 28.2/h. At a mean follow-up of 5.0 years, the weighted postoperative AHI was 11.1 ± 13.0/h. Only one study [42], with 53 patients, reported long-term follow-up ESS (10.8 ± 5.0 to 10.2 ± 5.1, *p* > 0.05). Boyd et al. [30] reported a long-term improvement in FOSQ score after MMA. Surgical success rate was reported in two studies [38,46] (90% and 41.4%, respectively), and surgical cure rate was only available in one study [42] (67.9%).

UAS group. Three studies [15,50,51] reported long-term follow-up (≥2 years) data in 127 patients with weighted baseline AHI of 29.7 ± 11.0/h. At a mean follow-up of 4.2 years, the weighted postoperative AHI was 12.3 ± 14.8/h. These three studies [15,50,51] also reported a long-term improvement in ODI and ESS after UAS therapy. One study [15] reported a long-term (five years follow-up) improvement in FOSQ score. Surgical success and cure rates were reported in all three studies [15,50,51] (success rate: 77.8%, 71.1%, and 74.6%, respectively; cure rate: 33.3%, 35%, and 44%, respectively).

### 3.7. Safety

There were no studies reporting any deaths related to MMA or UAS surgery.

MMA group. Of the included studies, 10 reported participants’ complications after MMA (n = 428) [28,30,33,39,42,43,44,45,46,47]. The rate of major complication ranged from 0 to 18%. Five studies reported the major compilations after MMA, which included reoperations for removal of osteosynthesis screws and plates (n = 8) [30,33,46], reoperations for maxillary non-union (n = 2) [28,46], and acute dyspnea (n = 1) [45].

The most common minor complication reported was facial paresthesia caused by the impairment of inferior alveolar nerve [30,33,39,43,45,46,47]. Four studies [39,45,46,47] reported both the rates of transient and persistent paresthesia in mandibular area, which were 100% and 13% (n = 175), 100% and 28% (n = 25), 90% and 60% (n = 34), and 32% and 0% (n = 28), respectively. Additionally, one study [43] (n = 34) reported only the rate of transient paresthesia in mandibular area—75%; one study [33] (n = 11) reported only the rate of the persistent symptom—27%. In the long-term follow-up study from Boyd et al. [30] (n = 30), although no patients exhibited such facial anesthesia as measured objectively, 40% of patients subjectively perceived a decrease in sensation. Facial paresthesia in the infraorbital area was reported by two studies [45,46]. In the study by Vicini et al. [45] (n = 25), the rates of transient and persistent paresthesia in infraorbital area were 100% and 4%, respectively; in the study by Vigneron et al. [46] (n = 34), they were 37% and 30%, respectively.

Excluding facial paresthesia, the other reported minor complications consisted of developed malocclusion [30,45,46,47] (n = 13), temporomandibular disorders [46,47] (n = 11), local infection [28,30,47] (n = 6), minor postoperative wound pain [33] (n = 2), and others (n = 5) [28,44,47]. Of ten studies [28,30,32,41,42,43,44,45,46,47] that investigated patients’ perception of their facial appearance after MMA, two studies [30,46] reported that there were 13% (4/30) and 15% (5/34) patients who perceived worsening of their facial appearance after MMA, respectively; the others [28,32,41,42,43,44,45,47] reported that the perception of facial appearance was positive or neutral in all the patients after MMA.

UAS group. Of the five studies reporting patients’ complications (n = 2051) [15,49,51,52,54], the rate of serious device-related adverse events range from 0 to 7%. Four studies [15,51,52,54] reported a total of 50 serious device-related adverse events requiring surgical repositioning or replacement of the neurostimulator or implanted leads. In addition, in the study from Suurna et al. [54] (n = 1849), 0.4% of the patients reported serious intraoperative adverse events, including but not limited to hematoma (n = 8), infection (n = 2), extra implant procedure (n = 1), intraoperative arrest (n = 1), and pneumothorax (n = 1).

Since one study [54] did not report the count of minor complications, the safety outcomes of a subset of the study population (ADHERE cohort) reported in a previous study [56] were used to analyze the minor complication rate. In that study [56], the rates of minor surgery-related and device-related complications 137 ± 77 days after UAS implant were 6% (18/313) and 22% (69/313), respectively; 386 ± 136 days after UAS implant were 4% (8/217) and 24% (53/217), respectively. In the STAR trial cohort [15] consisting of 126 participants, the rates of minor surgery-related and device-related complication were both 136% (171/126) at the first year; at the fifth year, they were decreased to 1% (1/126) and 16% (20/126), respectively. Van de Heyning et al. [52] reported only minor surgery-related adverse events in their population, which yielded a minor complication rate of 57% (16/28). Philip et al. [49] and Steffen et al. [51] did not report any minor complications in their study populations. The most common minor surgery-related and device-related complications were incision discomfort [15,51,56] and discomfort due to electrical stimulation [15,56], respectively.

## 4. Discussion

This is the first systematic review aiming to comparatively evaluate MMA and UAS therapy in treating OSA. We reviewed 21 studies on MMA and 9 studies on UAS in treating OSA. Due to the fact that there is no RCT or comparative study of MMA and UAS, a meta-analysis cannot be performed to directly compare these two interventions. Separate analyses of studies on MMA and UAS were utilized for this review. In this review, the trials for MMA tended to be published earlier than those for UAS. Therefore, for some patients in the UAS group, MMA could have been considered at first as an alternative therapy to CPAP and not been chosen. It should be noted that UAS therapy has stricter and clearer inclusion criteria (e.g., 15/h ≤ AHI ≤ 65 /h, absence of CCCp during DISE) [14,17] for patients, especially in comparison to MMA. There is therefore discrepancy of patients’ baseline characteristics between the MMA cohort and UAS cohort. In this review, the MMA cohort has younger age and higher baseline AHI compared to the UAS cohort. Moreover, it is impossible for us to compare other patients’ characteristics associated with OSA, such as the size of tongue, retrolingual space, and jaw position. To obtain definitive results on the comparison of MMA and UAS, future studies should include comparative studies of these two therapies where participants would have comparable baseline characteristics and be qualified for both therapies. Another point to be noted is that the variations in MMA surgeries are probably greater than in UAS as the training and the lineage of potential variations are much higher in MMA than in UAS.

### 4.1. Objective Outcomes

Based on the separate analysis of studies on MMA and UAS, we reported that these two procedures are both effective treatment modalities for OSA. However, compared to UAS, MMA seems to be more effective in treating OSA with a more significant decrease in AHI and higher success rate. Through different mechanisms, MMA and UAS have been proven to be able to address multiple sites of collapse simultaneously [11,36]. MMA enlarges the entire pharynx and reduces the collapsibility of the upper airway by advancing the maxillomandibular complex and anterior pharyngeal tissues attached to the maxilla, mandible, and hyoid bone [39]. The mechanism by which UAS resolves multilevel collapse, is enlargement of the retropalatal airway associated with tongue protrusion, which is so called “palatoglossus coupling” phenomenon [48]. Safiruddin et al. found that the retropalatal enlargement in response to UAS was statistically significant only in the responders, while the responders and non-responders had similar degrees of retrolingual opening to stimulation [57]. Therefore, we are of the opinion that the superiority of MMA over UAS in OSA treatment may be associated with the ability of MMA to enlarge the retropalatal airway more significantly. To improve patient selection for MMA and UAS, the mechanism of action of these two surgical procedures and the role of pathogenesis of OSA on the outcome of both surgeries require clarification in future studies.

### 4.2. Subjective Outcomes

It is interesting to note that several studies [42,55] reported a discordance between objective outcome measures (e.g., AHI) and patient-reported outcome measures, which highlights the importance of subjective outcome evaluation for OSA patients. In contrast to published ESS data, there is a scarcity of evidence related to other subjective outcomes of surgical treatment for OSA. Boyd et al. [30] evaluated the impact of MMA on quality of life (QoL) using the Functional Outcomes of Sleep Questionnaire (FOSQ). Two years after MMA, a significant improvement in mean FOSQ scores of 4.7 was observed. In a study by Woodson et al. [15], the improvements in mean FOSQ scores following UAS were 3.0 at 1 year and 3.7 at 5 years, respectively. In addition to daytime sleepiness and QoL, patient satisfaction—an important measure of therapy quality—should be noted when evaluating treatment options for OSA. Currently, only a few studies have evaluated patient satisfaction with MMA or UAS for the management of OSA [56,58,59,60,61,62]. In a study by Butterfield et al. [59], 95.5% of patients were satisfied with MMA surgery for OSA, 90.9% would repeat the procedure, and 86.4% would recommend MMA to others for OSA treatment. In the ADHERE registry, 94% of patients reported that they were satisfied with UAS therapy and would undergo UAS again, and 93% reported that they would recommend UAS to others [56]. According to the available evidence, both MMA and UAS could significantly improve the perception for OSA patients with high levels of patient satisfaction. However, the comparison of improvement in patient-perceived measures between the two therapies must be addressed in future studies.

### 4.3. Long-Term Outcomes

The long-term follow-up period of the included MMA studies ranges from 2 years to 12.5 years. Because of the small sample size, one study by Pottel et al. [63] reporting the longest follow-up result of MMA was excluded. In that study, the short term (within 2 years) success rate was 66.67% (8/12), and the long-term (median 19 years; range 14–20 years) success rate of MMA was 44.44% (4/9). Of the nine patients who attended long-term re-evaluation, the median ages at the time of MMA surgery and re-evaluation were 43 years (range 34–63 years) and 62 years (range 49–82 years), respectively. At the long-term follow up, two of the six patients who were initially successfully treated by MMA had relapse of OSA with AHI comparable to preoperative values. Both patients had significant weight gain (+4.1 and +7.9 kg/m^2^). In a study of 29 OSA patients treated by MMA, Vigneron et al. [46] concluded that the success rate was 85.7% in the immediate postoperative period and 41.1% at 12.5 years. Additionally, they concluded that the good candidates for long-term success of MMA were the young patients (<45 years old) with BMI < 25 kg/m^2^, AHI < 45/h, SNB angle < 75°, narrow retrolingual space (<8 mm), preoperative orthodontics, and without co-morbidity. It has been suggested that long-term failure of MMA might be attributed to weight gain [38,63,64], skeletal relapse [64], and ageing [63]. Given that UAS is an innovative therapy for OSA from the last decade, the longest follow-up period of the UAS studies was 5 years, from the STAR trial [15]. The success rates of UAS in the STAR trial cohort were 66% (83/126), 74% (73/98), and 75% (53/71) at 1, 3, and 5 years, respectively. In UAS therapy for OSA treatment, patients’ adherence is necessary to guarantee clinical efficacy [65]. The STAR trial revealed a high adherence to UAS therapy in the long-term, with a patient-self-reported nightly device use of 80% at 5 years, which might partially explain the stability of treatment effect. In addition, lower baseline ODI was found to be predictive of 5-year response to UAS therapy. It is therefore concluded that both MMA and UAS were relatively stable treatments for patients with moderate-to-severe OSA. In order to maintain clinical efficacy, more effort is needed to provide continuous follow-up for OSA patients and to ascertain the factors associated with long-term stability of outcomes.

### 4.4. Safety

In terms of treatment safety, this systematic review revealed that both MMA and UAS were generally safe surgical procedures for OSA, with relatively low rates of major complication. In the included MMA studies, all but one of the major complications were reoperation for removal of hardware. Age has been shown to be a risk factor for increased need for hardware removal [66]. In addition, Passeri et al. found that patients who were active smokers or had a history of smoking had higher risk of complications, which included removal of hardware [67]. The most common minor complication of MMA detailed in the literature was paresthesia of the lower lip and chin. It has been suggested that age at the time of surgery and addition of a genioplasty increase the risk of facial paresthesia, and a large degree of advancement further increases the risk in older patients [68,69]. In the STAR cohort (n = 126), the rates of major complication requiring device explanation, reposition, or replacement were 4% at 4 years and 9.5% at 5 years, indicating that the reoperations after UAS may occur more often during the late time frame. The STAR cohort also suggested that the majority of minor complications after UAS were gradually resolved. Notably, Withrow et al. evaluated the impact of age on safety of UAS and found no significant difference between younger and older cohorts in complication rates [70]. Current evidence suggests that both MMA and UAS appear to be safe approaches in OSA treatment, and compared to MMA, treating OSA with UAS may lead to fewer complications for older patients.

### 4.5. Clinical Relevance

In patients with moderate to severe OSA and failure of CPAP treatment, a portion of them could qualify for both MMA and UAS therapy. Current evidence shows that MMA may have superior efficacy in OSA treatment. However, MMA is a more invasive intervention, exposing patients to longer recovery time and higher risk of postoperative complications. Overnight admission to the intensive care unit is required for OSA patients following MMA surgery, and the length of hospitalization after MMA reported previously ranged from <2 days to 5–8 days [69]. Additionally, MMA surgery often involves time-consuming preoperative and/or postoperative orthodontic work. One notable potential problem with MMA has been the accompanying alteration in facial appearance; however, most patients undergoing MMA for OSA view the change in facial appearance as neutral or even positive [30,32,46]. In comparison to MMA, UAS surgery is less invasive and more patient-friendly and does not require extended recovery. The majority of patients are discharged the same day or one day after UAS surgery [71]. In addition to the information regarding treatment efficacy and safety, the cost of treatment options is important in assisting decision-making in OSA treatment. It has been indicated that UAS is cost-effective, with a lifetime incremental cost effectiveness ratio (ICER) of USD 39,471 per quality-adjusted life year (QALY) in the United States healthcare system [72] and EUR 44,446 per QALY in a European setting [73]. However, to our knowledge, no study has assessed the cost-effectiveness of MMA, which precludes the comparison of cost-effectiveness between these two therapies. Hence, to further assist decision-making in OSA treatment, there is a need to assess and compare the costs and cost-effectiveness of each intervention.

Since the primary target patient population differs between MMA and UAS, these two procedures are usually not put on par in the current practice guidelines. In the current Stanford protocol, UAS and MMA are considered phase I and phase II surgical procedures, respectively [74]. It has been proposed that these two procedures might be considered as complementary therapies [17]. For example, UAS may be considered when a patient fails to respond to MMA or for a patient with relapse of OSA after previously successful MMA [75]. It is interesting to note that in a recent study [76], Sarber et al. evaluated the efficacy of UAS therapy in 18 OSA patients who did not meet all FDA criteria for UAS and found promising treatment outcomes. They suggested that future studies must consider the expansion of current FDA criteria for UAS, particularly in BMI and AHI criteria. Thus, to optimize surgical outcomes, reduce rates of mortality and morbidity, and improve quality of life and other subjective outcomes, further investigation is essential to clarify indications of each therapy for OSA.

In addition to MMA and UAS, there are other evidence-based therapeutic options for OSA, which include behavioral strategies (e.g., weight loss), medical therapy (e.g., CPAP), other surgical options, and adjuvant therapy (e.g., pharyngeal muscle training) [77,78]. Of the non-CPAP therapies for OSA, more invasive procedures, such as MMA, are not well accepted. Oral appliances offer a non-invasive option for managing OSA, the most common of which are mandibular advancement devices (MADs). MADs modify the position of the jaw, the tongue, and other supporting structures of the upper airway, thereby increasing upper airway volume and preventing collapse of the upper airway [79]. MADs are recommended as a first-line therapy for mild-to-moderate OSA and for severe OSA after CPAP failure, intolerance, or refusal [80]. Growing evidence suggests that MADs could achieve favorable outcomes regardless of the severity of OSA [81,82].

In the era of precision medicine, the interconnected risk factors for OSA must be considered in order to achieve precision medicine in OSA [78]. The combined modern therapies for OSA must be adjusted continuously in respect to recent scientific research in order to deliver the best results for patients, emphasizing their quality of life in addition to medical care. Therefore, any of the therapies may either have an important role as monotherapy in the treatment of OSA or could be used in combination with the other therapies. The greater the complexity of a clinical case, the greater the need for multidisciplinary collaboration.

### 4.6. Limitations

There are several limitations of the present review. Firstly, because of the inherent difficulty of randomizing patients to different surgical interventions or sham surgery [83], except for one RCT and one quasi-experimental trial, all the included studies were cohort studies, the majority of which demonstrated fair quality according to the MINORS tool. Due to the lack of RCT and comparative studies of MMA and UAS for OSA, a meta-analysis cannot be performed to directly compare these two procedures. Additionally, meta-analyses were not conducted to separately assess overall effect sizes of MMA and UAS therapy on OSA, as mean and SD of the difference between pre- and postoperative measures were absent in majority of the selected studies. In this review, we performed separate analyses for MMA and UAS studies, combined with noticeable differences between the two cohorts in age and OSA severity, which prevented us from generating a solid conclusion on the comparison of these two procedures. Due to the fact that some patients may fall between two stools, comparison of the two procedures is important. Future studies should include quasiexperimental trials and comparative cohort studies comparing MMA and UAS to better clarify which modality is superior in OSA treatment. These studies can be part of a future large international consortium, which is more likely to generate solid conclusions. Secondly, due to the implemented inclusion criteria, which included the presence of both preoperative and postoperative PSG data, some well-conducted studies reporting on only subjective outcomes and/or safety were excluded for this study. Therefore, the present analysis of subjective outcomes and safety may not be entirely representative of the population undergoing MMA or UAS in the current literature. Lastly, our review is exclusively based on studies published in English, which can introduce a language bias [84].

## 5. Conclusions

The results presented in this review suggest that both MMA and UAS are effective and generally safe surgical treatment modalities for patients with moderate-to-severe OSA. However, within the limitation of the selected studies, there is currently no evidence on the comparison of MMA and UAS in the treatment of OSA.

## Data Availability

Not applicable.

## Supplementary Materials

The following supporting information can be downloaded at: https://www.mdpi.com/article/10.3390/jcm11226782/s1, Table S1: (a). Search strategy in MEDLINE database. (b) Search strategy in Embase database; Table S2: (a) Methodological appraisal of the individual studies according to MINORS assessment tool—maxillomandibular advancement surgery. (b) Methodological appraisal of the individual studies according to MINORS assessment tool—upper airway stimulation.
