# Supplementary material for: Maxillomandibular Advancement and Upper Airway Stimulation for Treatment of Obstructive Sleep Apnea: A Systematic Review
Source: J Clin Med. 2022 Nov 16;11(22):6782. doi: 10.3390/jcm11226782 (PMC9697253; doi:10.3390/jcm11226782)
Supplement: Supplementary file 1 [file jcm-11-06782-s001.zip › jcm-2010138-supplementary.pdf]

Supplementary Materials

Table S1 (a). Search strategy in MEDLINE database. (b) Search strategy in Embase database

**(a) Ovid MEDLINE(R) ALL <1946 to Dec 14, 2021>**		
**Step**	**Search**	**Result**
1	exp Sleep Apnea Syndromes/ or Snoring/ or ((sleep adj3 (apnea or apnoea or hypopnea or hypopnoea)) or (upper adj airway adj resistance) or (sleep adj disordered adj breathing) or snore or snoring).ti,ab,kf,ot.	54852
2	(mma or ((maxillomandibular or mandibular) adj2 advancement) or ((bimaxillar or orthognathic) adj2 surgery) or maxillary-osteomy or (multilevel or multi-level)).ti,ab,kf.	51520
3	'Electric Stimulation Therapy'/ or 'Electric Stimulation'/ or 'implantable Neurostimulators'/ or (((hypoglossal-nerve* or nervus-hypoglossus or cranial-nerve* or (XII adj nerve*)) adj2 (stimulat* or surgery or therap*)) or (upper-airway adj stimulat*) or Neurostimulat* or (implantable-nerve adj stimulat*) or electrical-stimulat*).ti,ab,kf.	52389
4	2 or 3	103811
5	1 and 4	1972
**(b) Embase Classic+Embase <1947 to Dec 14, 2021>**		
**Step**	**Search**	**Result**
1	exp 'snoring'/ or exp 'sleep disordered breathing'/ or (sleep adj3 (apnea or apnoea or hypopnea or hypopnoea)).ti,ab. or 'upper airway resistance'.ti,ab. or 'sleep disordered breathing'.ti,ab. or snor*.ti,ab.	97390
2	(mma or ((maxillomandibular or mandibula) adj2 advancement) or bimaxillar-surgery or maxillary-osteomy or orthognathic-surgery).ti,ab,kw.	8129
3	(multilevel or multi-level).ti,ab,kw.	49506
4	exp electrostimulation/ or exp 'nerve stimulator'/	101629
5	(((hypoglossal-nerve* or nervus-hypoglossus or cranial-nerve* or (XII adj nerve*)) adj2 (stimulat* or surgery or therap*)) or (upper-airway adj stimulat*) or Neurostimulat* or (implantable-nerve adj stimulat*) or electrical-stimulat*).ti,ab,kw.	71405
6	2 or 3 or 4 or 5	190441
7	1 and 6	2037
8	(exp experimental organism/ or animal tissue/ or animal cell/ or exp animal disease/ or exp carnivore disease/ or exp bird/ or exp experimental animal welfare/ or exp animal husbandry/ or animal behavior/ or exp animal cell culture/ or exp mammalian disease/ or exp mammal/ or exp marine species/ or nonhuman/ or animal.hw.) not human/	7698484
9	7 not 8	1960
10	limit 9 to (conference abstracts or embase)	1775

Table S2. (a) Methodological appraisal of the individual studies according to MINORS assessment tool – maxillomandibular advancement surgery. (b) Methodological appraisal of the individual studies according to MINORS assessment tool – upper airway stimulation

**(a)**														
	Q1	Q2	Q3	Q4	Q5	Q6	Q7	Q8	Q9	Q10	Q11	Q12	Total score	Quality
Bettega et al. 2000 [28]	2	2	0	2	1	2	2	0					11	Fair
Bianchi et al. 2014 [29]	2	2	0	2	1	2	0	0					9	Fair
Boyd et al. 2015 [30]	2	2	2	2	1	2	0	2					13	High
Conradt et al. 1997 [31]	2	2	0	2	1	2	2	0					11	Fair
Gerbino et al. 2014 [32]	2	2	2	2	1	2	2	0					13	High
Goh et al. 2003 [33]	2	2	2	2	0	2	2	0					12	Fair
Goodday et al. 2016 [34]	2	2	0	2	0	2	0	0					8	Fair
Hsieh et al. 2014 [35]	2	0	2	2	1	2	0	0					9	Fair
Kastoer et al. 2019 [36]	2	0	2	2	1	2	2	0					11	Fair
Li et al. 1999 [39]	0	2	0	2	0	2	2	0					8	Fair
Li et al. 2000 [38]	2	2	0	2	0	2	0	0					8	Fair
Li et al. 2001 [40]	2	2	0	2	0	2	0	0					8	Fair
Li et al. 2002 [37]	2	1	2	2	0	2	0	0					9	Fair
Liao et al. 2015 [41]	2	2	2	2	1	2	0	0					11	Fair
Lin et al. 2020 [42]	2	2	2	2	1	2	0	0					11	Fair
Liu et al. 2016 [11]	2	2	0	2	1	2	0	0					9	Fair
Rubio-Bueno et al. 2017 [43]	2	2	2	2	1	2	0	0					11	Fair
Veys et al. 2017 [44]	2	2	2	2	0	2	0	0					10	Fair
Vicini et al. 2010 [45]	2	2	2	2	1	2	2	0					13	High
Vigneron et al. 2017 [46]	2	2	0	2	1	2	0	0					9	Fair
Wu et al. 2019 [47]	2	2	0	2	1	2	0	0	0	2	0	2	13	Fair

(b)										
	Q1	Q2	Q3	Q4	Q5	Q6	Q7	Q8	Total score	Quality
Bachour et al. 2021 [55]	2	2	0	2	0	2	2	0	10	Fair
Heiser et al. 2017 [48]	2	2	2	2	0	2	0	0	10	Fair
Philip et al. 2018 [49]	2	0	2	2	1	2	0	0	9	Fair
Steffen et al. 2019 [50]	2	2	0	2	0	2	0	0	8	Fair
Steffen et al. 2020 [51]	2	2	2	2	0	2	0	0	8	Fair
Suurna et al. 2021 [54]	2	2	2	2	0	2	0	0	10	Fair
Van de Heyning et al. 2012 [52]	2	2	2	2	1	2	2	0	13	High
Vanderveken et al. 2013 [53]	2	0	2	2	1	2	0	0	9	Fair
Woodson et al. 2018 [15]	2	0	2	2	1	2	0	0	9	Fair

Q1, a clear study aim; Q2, inclusion of consecutive patients; Q3, prospective collection of data; Q4, endpoint appropriate to the aim of the study; Q5, unbiased assessment of the study; Q6, follow-up period appropriate to the aim of the study endpoint; Q7, loss of follow-up less than 5%; Q8, prospective calculation of the study size; Q9, an adequate control group; Q10, contemporary group; Q11, baseline equivalent of groups; Q12, adequate statistical analysis.
